# Ultrafast Band Engineering and Transient Spin Currents in Antiferromagnetic Oxides

**DOI:** 10.1038/srep25121

**Published:** 2016-04-29

**Authors:** Mingqiang Gu, James M. Rondinelli

**Affiliations:** 1 Department of Materials Science and Engineering, Northwestern University, Evanston, IL 60208, USA

## Abstract

We report a dynamic structure and band engineering strategy with experimental protocols to induce indirect-to-direct band gap transitions and coherently oscillating pure spin-currents in three-dimensional antiferromagnets (AFM) using selective phononic excitations. In the Mott insulator LaTiO_3_, we show that a photo-induced nonequilibrium phonon mode amplitude destroys the spin and orbitally degenerate ground state, reduces the band gap by 160 meV and renormalizes the carrier masses. The time scale of this process is a few hundreds of femtoseconds. Then in the hole-doped correlated metallic titanate, we show how pure spin-currents can be achieved to yield spin-polarizations exceeding those observed in classic semiconductors. Last, we demonstrate the generality of the approach by applying it to the non-orbitally degenerate AFM CaMnO_3_. These results advance our understanding of electron-lattice interactions in structures out-of-equilibrium and establish a rational framework for designing dynamic phases that may be exploited in ultrafast optoelectronic and optospintronic devices.

Light-matter interactions can be utilized to induce ultrafast phenomena in correlated electron materials^1^ for realizing functionalities that can neither be observed in bulk equilibrium^2^,^3^,^4^,^5^ nor by means of static perturbations to the structure, including chemical substitution, mechanical strain, or digital heterostructures. Inducing nonequilibrium phonon modulations are of particular recent interest owing to their direct^6^ and indirect^7^,^8^,^9^,^10^ accessibility with high intensity femtosecond laser pulses ranging from the mid-infrared (IR) to terahertz regimes^11^. This athermal light-enabled structure–property control, for example, has been exploited in the superconducting cuprate family, where the electron pairing strength is related to the Cu-O intra-bilayer distance^12^: Mid-IR pulses are able to induce transient high-*T*_*C*_ superconductivity from a nominally non-superconducting material^13^ or melt charge order in a superconducting cuprate at subpicosecond timescales^14^ by direct excitation of IR-active Cu–O bond stretching modes. Recently, ionic Raman scattering (IRS) has also been used to dynamically activate Raman modes by leveraging anharmonic interactions with a pumped IR mode leading to in-plane buckling of the lattice planes in bi-layer cuprates and enhanced superconducting transport^15^,^16^.

## Results

### Dynamical Structure–Band Gap Design Principles

**Figure 1:**
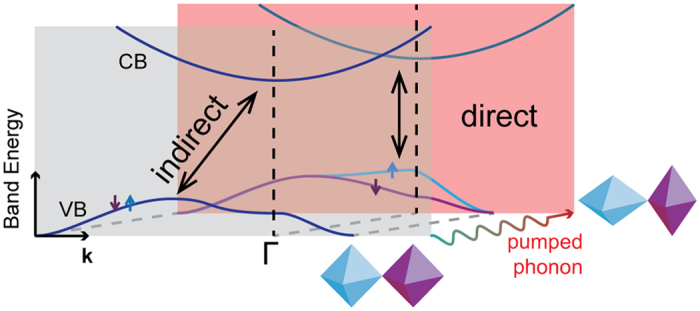
Dynamical modulation of transition-metal–oxygen polyhedra enables band gap control in antiferromagnetic oxides. Schematic illustration depicting how a nonequilibrium amplitude of a normal mode that leads to a shape change in the *B*O_6_ polyhedra can both reduce the electronic band gap and lift the spin degeneracy through light-induced electron-phonon interactions. Spin up and spin down channels are shown in blue and purple, respectively. The polyhedral coloring (inset) shows that the phonon modulation vector is identical to the magnetic ordering.

Here we establish dynamical materials design principles for light-induced phononic band structure control in a ubiquitous transition metal oxide family exhibiting antiferromagnetic (AFM) spin order. In collinear *G*-type AFM insulating oxides with the perovskite *AB*O_3_ structure, the *B* cations are located within corner-connected *B*O_6_ octahedra and the *B*-site transition metal ions form a three-dimensional simple cubic lattice with *d*^*n*^ spin up and spin down electrons alternating from site to site. In centrosymmetric materials at equilibrium, the two spin channels are degenerate with band energies *E*(*k*, ↑) = *E*(*k*, ↓). Owing to crystal point symmetries, some normal modes are able to modify the equivalency of the two spin sublattices^17^, and therefore can lift the spin degenerate band structures defining the AFM state (Fig. 1). But how does one coherently drive an indirect-to-direct band gap transition by coupling of the electronic (spin) structure to the lattice through a photon?

To establish those guidelines, we first recognize that the effect of exciting a normal mode is to change the eigenenergies for the two spin channels at the reciprocal space lattice vector satisfying 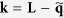 (Criterion 1), where **k** is the difference in the modulation vectors for the antiferromagnetism (**L**) and phonon 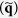. (Note that we use 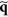 for the local modulation vector, distinguishing it from the phonon propagation wave vector **q**). If the two modulation vectors are identical 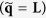, then the primary changes to the eigenenergies will be at Γ. Criterion 1 does not specify the symmetry of the phonon mode for excitation. Rather, it is determined when one tries to ‘match’ the normal mode modulation vector to the magnetic propagation vector. If the magnetic point group is polar, for example, then the targeted normal mode is likely to be Raman and IR active, whereas in centrosymmetric magnetic groups only Raman modes will satisfy 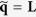. These details further specify the experimental route by which one would achieve the quasi-static nonequilibrium displacements: IR-active modes may be driven coherently by applying a pulse with the same frequency; in contrast, coherent Raman pumping requires more sophisticated stimulation methods, such as impulsive stimulated Raman scattering^18^ (ISRS) or coherent anti-Stokes Raman spectroscopy (CARS)^19^. Compared to direct IR mode excitations, ISRS has the advantage that such a process is off-resonance and thus brings flexibility in the selection of the pump frequency. The frequency usually ranges from 200–500 THz^18^,^20^. Although the higher frequency might lead to photoelectron or quasiparticle excitations, in our case these can be reduced by choosing an appropriate photon frequency as discussed further below.

Although Criterion 1 guarantees a modification in the band energies, it does not require that substantial perturbations to the equilibrium energies occur—even for highly nonlinear displacements of the normal mode. We propose that substantial control over the eigenenergies relies on strong electron-lattice coupling (Criterion 2). The correlated nature of an electronic system near an ordered state would ensure strong spin-orbital-lattice responses with respect to the quasi-static displacement of the excited normal mode. This guideline allows us to downselect from the set of symmetry suitable normal modes provided by Criterion 1 to a targeted phonon, which produces bond length or angle changes that directly affect the states near the Fermi level. As we show next, a Raman active mode (*B*_2*g*_ symmetry) pervasive in orthorhombic perovskite oxides, which describes a first-order Jahn-Teller distortion (FOJT), fulfills both criteria.

In this next section, we apply our design principles to two strongly-correlated perovskites with the same magnetic ordering vector to explore the dynamical structure–property relationships under phononic excitation. We first apply these principles in detail for LaTiO_3_, explaining the origin of the indirect-direct band gap transition in AFM insulators. Then we show how polarized spin currents are generated in correlated metals (hole-doped LaTiO_3_) and also apply the approach to orbitally degenerate CaMnO_3_. Finally we show how to use ISRS to induce the response.

### Application to LaTiO_3_

#### Equilibrium Structure and Properties

**Figure 2:**
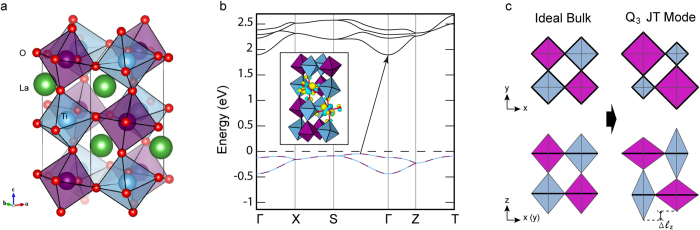
Equilibrium structure and electronic ground state of LaTiO_3_. Crystal structure (**a**) and electronic band structure (**b**) of orthorhombic LaTiO_3_, highlighting the Ti *t*_2*g*_ states. The valence band maximum is located between the S and Γ points in the lower Hubbard band (below the dashed horizontal line) while the conduction band minimum in the upper Hubbard band is at the Γ point. The spin up and down Ti sites are denoted with blue and purple coloring, respectively. Owing to the spin degeneracy in the AFM state, the majority and minority bands overlap each other as indicated by the solid (blue, spin up) and broken (purple, spin down) lines in the valance band in (**b**). The inset in (**b**) shows the MLWF for the occupied t_2g_ orbital with weak orbital ordering. (**c**) Schematic picture of the FOJT distortion corresponding to the *Q*_3_ mode.

LaTiO_3_ is a correlated Mott insulator (space group *Pbnm*) with a *G*-type antiferromagnetic (AFM) order (Fig. 2)^21^. The measured indirect optical semiconducting gap is Δ ~ 0.1 to 0.2 eV^22^. LaTiO_3_ is host for strong electron-lattice interactions owing to the 3*d*^1^ electronic configuration of Ti^3+^, which makes the spin and orbital degrees of freedom highly sensitive to the lattice phonons, including those describing local bond distortions and TiO_6_ octahedral rotations^23^. Indeed, model calculations show that the FOTJ distortion splits the *t*_2*g*_ orbitals and leads to an orbital polarization in the system^24^, supporting the concept that FOJT modulations could be utilized to achieve a nonequilibrium electronic phase.

Figure 2b shows the equilibrium electronic structure for LaTiO_3_ obtained using density functional theory (DFT) within the generalized-gradient approximation plus Hubbard *U* approach (see Methods). Consistent with prior theoretical^23^ and nuclear magnetic resonance (NMR) studies^21^, we find the *G*-type AFM order is the lowest energy spin configuration. The material is also semiconducting with an indirect band gap of 1.89 eV, which is larger than that measured experimentally. (We discuss the role of the *U* correction on the gap size discrepancy in the Methods section, but note that the overestimate does not lead to any qualitative differences in the *nonequilibrium* physics). The valance band maximum (VBM) is located between the Γ and *S* points and the conduction band minimum (CBM) is at Γ.

The Ti-*t*_2*g*_ manifold plotted in Fig. 2b is split by the on-site Coulomb repulsion into two Hubbard bands with 0.39 eV (lower Hubbard) and 0.78 eV (upper Hubbard) bandwidths, respectively. A weak orbital ordering is realized among the three *t*_2*g*_ orbitals in the lower Hubbard band owing to partial occupancy as seen from the maximally localized Wannier functions (MLWF) plotted in the inset: This is confirmed by integrating the *t*_2*g*_ density of states (DOS). The occupation number *n* for the majority and minority spin in-plane *d*_*xy*_ orbital is 0.24*e*, while the occupancies for the out-of-plane *d*_*yz*_ and *d*_*zx*_ orbitals are spin-dependent, alternating in magnitude to preserve the AFM state. For the majority spin electrons, *n*(*d*_*yz*_) = 0.31 and *n*(*d*_*zx*_) = 0.11, while for the minority spin channel, *n*(*d*_*yz*_) = 0.11 and *n*(*d*_*zx*_) = 0.31. It is clear that an alternating orbital polarization, identical to the AFM spin order, occurs among nearest neighbor Ti atoms in the equilibrium state. Critically, this weak orbital order is obtained even without spin-orbit coupling (SOC)—the low-energy band structure depends on the local atomic structure, which contains a static FOJT with Ti–O bond length differences of 
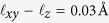
.

#### Normal Mode Selection

We now search for a normal mode that fulfills the two postulated criteria in LaTiO_3_. The zone-center normal modes, including 25 IR-active modes and 24 Raman modes are computed at the DFT + *U* level (see Methods). The *Q*_3_ FOJT mode^25^ (Raman *B*_2*g*_ symmetry with a calculated frequency *ω* = 337 cm^−1^) is selected as follows: First, the character of the mode involves the displacement of two apical oxide anions that are out-of-phase with respect to the four in-plane oxide anions in a TiO_6_ unit. The three-dimensional behavior of the mode consists of adjacent octahedra distorting in the reverse sense of the FOJT mode, leading to alternating TiO_6_ octahedra with a 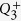 (elongation and contraction of two apical and four equatorial Ti-O bonds, respectively) and 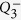 (vice versa) modulation as depicted in Fig. 2c. Here, the long range modulation vector for this ordering is 2*π*/(**l**_1_ + **l**_2_), where **l**_1,2_ = **a**, **b** or **c** with **a**, **b** and **c** the *B*-site sublattice vectors, giving a phase angle of 0° such that it is identical to the magnetic and orbital order 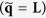. Owing to the 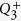 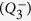 modulation in the majority (minority) spin channel, there is a corresponding volume reduction (expansion), which leads to an increase (decrease) in the *t*_2*g*_ band energies, respectively, from the ligand field effect.

#### Quasi-static Electronic Response

**Figure 3:**
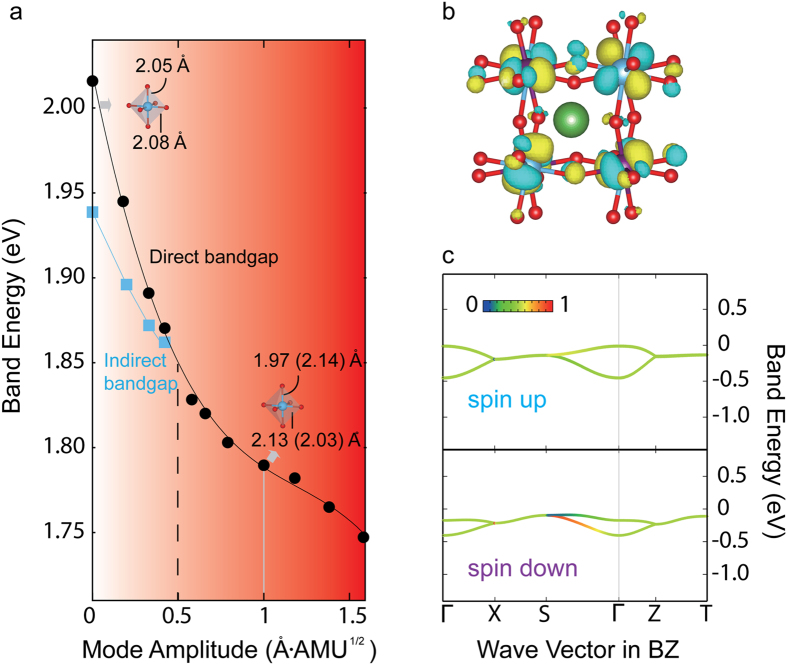
Origin of the indirect-to-direct band gap transition with nonequilibrium FOJT bond distortions. (**a**) The band evolution as a function of the *Q*_3_ normal mode amplitude. The broken vertical line shows the transition point at the critical *Q*_*c*_ ~ 0.5 Å AMU^1/2^. The insets in (**a**) indicate the change in Ti-O bond distances at different values of the mode amplitude. (**b**) The MLWFs for the lower Hubbard bands shows enhanced orbital ordering at *Q* = 1 Å AMU^1/2^. (**c**) The projected valence bands (color scale) on to the two *t*_2*g*_ orbitals (*d*_*xz*_ = 0 and *d*_*yz*_ = 1). The spin up valence band edge is at the Γ point.

We now examine a nonequilibrium LaTiO_3_ structure with varying amplitude of the FOJT mode with the assumption this such a local structural geometry is experimentally accessible as a quasi-static configuration. Figure 3 shows the electronic response to the *Q*_3_ normal mode: The main effect is a reduction in both the direct and indirect band gaps owing to changes at the VBM in the lower-Hubbard band. As the mode amplitude *Q*, which here is a summation of all atomic displacements present, increases the majority spin band energies at Γ shift to higher energies as the minority spin eigenenergies shift to lower energy. The change in the densities of state at the CBM, on the other hand, is negligible. At the critical value of *Q*_*c*_ ~ 0.5 Å (atomic mass unit)^1/2^ (or Å AMU^1/2^), we find that an indirect-to-direct band gap transition is activated. For a mode amplitude of 1 Å AMU^1/2^, the change in the apical (equatorial) Ti-O bond length is ±0.08 Å (±0.05 Å) as indicated in Fig. 3a; this set of displacements is sufficient to produce a direct band gap of 1.79 eV, which is 0.22 eV lower (11% reduction) relative to the equilibrium value and should be achievable through an impulsive optical phonon excitation.

Before proposing how to experimentally induce the critical mode amplitude, we first describe the origin of this light-induced band engineering by transferring the periodic space Bloch wave functions for the lower-Hubbard band into real space using the MLWF method. The most dramatic change is observed in the orbitally ordered state. Compared to the equilibrium state, the orbital ordering is enhanced. The occupancy of one of the t_2*g*_ orbitals is nearly zero in order to minimize the Coulomb interactions: For the spin up Ti, the in-plane orbital is quenched, *n*(*d*_*xy*_, ↑) = 0.06e, while for the spin down Ti one of the out-of-plane orbitals is quenched, *n*(*d*_*zx/yz*_, ↓) = 0.01*e*., alternatively. (Fig. 3b) clearly shows the in-plane and out-of-plane orbital polarization among the spin up and spin down sublattices, as well as the alternation between the two nearest spin-down Ti orbitals.

We reconcile the enhanced orbital order as follows: In the *d*^1^ system, the single electron in the *t*_2*g*_ orbitals forms a *π*-bond with the oxide anions in the TiO_6_ octahedra. A change in the Ti-O bond lengths and octahedra volume produces a charge redistribution within the *t*_2*g*_ manifold. The nature of the on-site charge transfer is sensitive to the phase of the *Q*_3_ modulation: For the majority spin channel (TiO_6_ undergoing 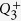), the *t*_2*g*_ electron is localized to the orbital aligned along the apical ligands whereas for the minority spin channel (TiO_6_ undergoing 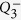) it occupies the equatorial orbitals, reflected in the MLFWs appearing in Fig. 3b. This change in the local orientation of the occupied orbital is required to reduce the contribution of the Coulomb energy in the *π*-bond, and this lifts the band energy spin degeneracies in the lower-Hubbard band at the VBM (Fig. 3c). Since this response requires the opposite modulation to the neighboring octahedra, it cannot be achieved by static (biaxial) strain (Supplementary Fig. 1); it is unique to the mode selective excitation.

#### Pure Spin Current in Hole-doped LaTiO_3_

Closure of the band gap through carrier doping by means of metal-insulator transitions have been observed in hole doped titanates^26^. Here we explore the effect of the quasi-static modulation on the metallic state of LaTiO_3_ with 0.25 holes per formula unit (mimicking La_3/4_Sr_1/4_TiO_3_). At equilibrium this level of hole doping gives a nonmagnetic metal owing to the delocalized carriers with the Fermi level located in the partially filled *t*_2*g*_ band.

**Figure 4:**
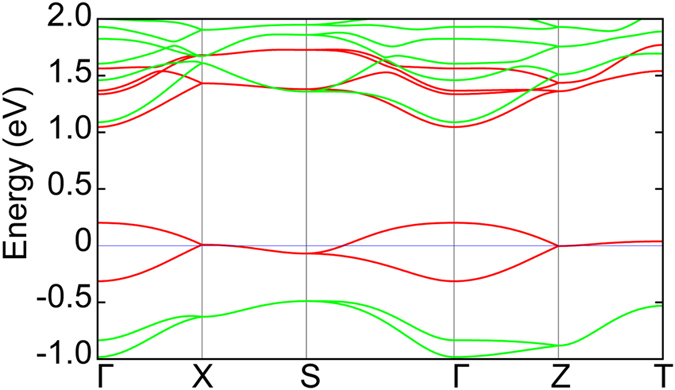
Band structure for hole-doped LaTiO_3_. At 1/4-hole doping (per formula unit) and a quasi-static phonon mode amplitude *Q* = 1 Å AMU^1/2^, the material becomes half-metallic with the Fermi level, horizontal (blue) line, crossing only the minority spin channel of the lower Hubbard band.

With a 1 Å AMU^1/2^ quasi-static displacement of the *Q*_3_ mode, corresponding to Ti–O bond length differences of 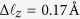, we find that the Fermi level intersects the lower Hubbard band of the minority spin channel (Fig. 4). Thus, a half-metallic ferromagnetic state emerges upon modulation of this mode. Furthermore, the minority and majority spin manifolds are separated by a well-defined energy gap of ~0.2 eV, which indicates that the phononic excitation should produce a pure spin current. Such a phononic activated 100% spin polarization should exceed that recently observed in doped silicon (50 ~ 80%)^27^,^28^.

### CaMnO_3_

#### Equilibrium Structure and Properties

**Figure 5:**
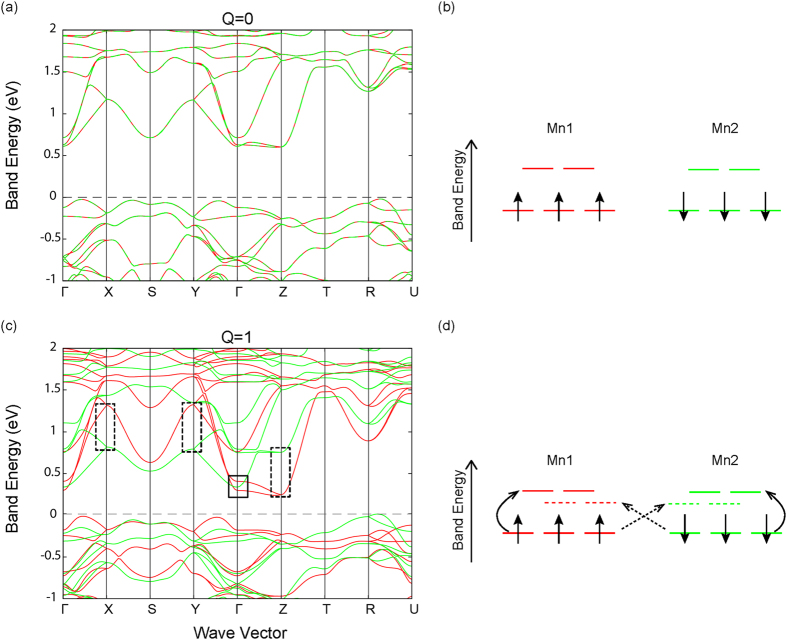
Band structure changes for CaMnO_3_ under modulation of the FOJT mode. The (**a**) equilibrium *Q* = 0 and (**c**) quasi-static *Q* = 1 Å AMU^1/2^ band structures projected on the two spin manifolds of the unique Mn cations. The spin degeneracy at the Γ point is lifted, denoted by the solid box in (**c**), under phononic excitation. The largest change in the band structure occurs at the **X**, **Y** and **Z** points at the CBM (denoted by the dashed boxes), rather than the VBM (denoted by the solid box). The idealized cubic crystal field for the Mn *d* orbitals splits into a 3-fold degenerate (*t*_2*g*_) and two-fold degenerate (*e*_*g*_) manifold for Mn sites 1 and 2 at (**b**) equilibrium and (**d**) with a quasi-static *Q*_3_ displacement. The *d*^3^ half-filling condition shifts the *e*_*g*_ orbital closer to the VBM in response to the *Q*_3_ mode. The arrows in (**d**) denote the on-site (solid) and inter-site (dashed) electronic transitions. The energy for the inter-site transition, which carries a wave vector at **X**, **Y** and **Z** points, is lowered due to the energy gained from the change in electrostatic potential with *Q*_3_.

CaMnO_3_ is isostructural to LaTiO_3_ (*Pbnm* space group) and exhibits *G*-type antiferromagnetic order. At equilibrium, we calculate that CaMnO_3_ has an indirect band gap of ~0.64 eV (with *U* = 5 eV), and a direct band gap at Γ of ~0.73 eV (Fig. 5a). Different from the *d*^1^ LaTiO_3_, CaMnO_3_ is a *d*^3^ system. Three electrons occupy the *t*_2*g*_ band, leading to a half-filling (Fig. 5b). Such orbital filling results in similar in-plane and out-of-plane Mn-O bond lengths, 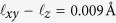 and effectively no orbital polarization among the Mn sites.

The similarity in crystal structure and magnetic ordering ensures that the same FOJT mode should enable band engineering. By calculating the phonon spectra at Γ point, we find the same FOJT *Q*_3_ mode is harder compared to LaTiO_3_ with a mode frequency of 584 cm^−1^.

#### Quasi-static Electronic Response

Figure 5c shows the band structure of CaMnO_3_ under excitation of the *Q*_3_ mode. The spin degeneracy at the Γ point is lifted by this mode. However, the effect at this wave vector is small compared to LaTiO_3_. With a mode amplitude of 1 Å AMU^1/2^, the direct band gap at the Γ point is reduced by 0.27 eV. Interestingly, unlike LaTiO_3_, now the largest change in the band structure occurs at the CBM rather than the VBM. Such differences between the two materials are attributed to the different electron filling of the *d* manifold. In CaMnO_3_ the majority spin channel comprised of all three *t*_2*g*_ orbitals is occupied. The lowest unoccupied orbital at the CBM is the *d*(3*z*^2^ − *r*^2^) orbital, which is of the same local symmetry of the *Q*_3_ FOJT mode. The 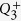 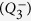 distortion lowers (increases) the electrostatic potential, and therefore the associated eigenenergies, of this orbital. The largest energy band splittings occur at non-zero *k*-vectors (at the **X** (1/2, 0, 0), **Y** (0, 1/2, 0) and **Z** (0, 0, 1/2) points, denoted by the dashed boxes in Fig. 5c), indicating that electron transitions to the nearest neighboring *d*(3*z*^2^ − *r*^2^) orbital along the *x*, *y* and *z* directions are energetically favored compared to the on-site transitions, schematically shown in Fig. 5d.

Although an indirect-to-direct band gap transition is not observed in CaMnO_3_, a large band gap reduction takes place at the **X**, **Y** and **Z** points due to the spin splitting in the CBM. For a mode amplitude of 1 Å AMU^1/2^, the reduction of the gap at these high symmetry points is ~0.5 eV, which is of a magnitude similar to that found for LaTiO_3_. Overall, CaMnO_3_ is one of many possible *G*-type AFM perovskites suitable for realizing dynamic band structure control as related vanadates, chromates, and ferrites, also fulfill the proposed criteria.

### Time-Dependent Structure–Property Relationships

#### Dynamics of the *Q*_3_ Mode and Electronic Properties

Now we take LaTiO_3_ as an example to explore the dynamical response in more detail. Owing to the high phonon frequency of the Raman-active *Q*_3_ mode, a displacive IRS process is unlikely to be feasible because it requires coupling a high frequency IR mode to an low frequency Raman mode. Therefore, we explore here how impulsive stimulated Raman scattering (ISRS) excites the *Q*_3_ mode. We consider a non-coherent process with the equation of motion^19^,^29^


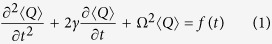


where *f*(*t*) is the impulsive driving force induced by the laser electric field, *γ* is the damping coefficient, 〈*Q*〉 and Ω are the Raman mode amplitude and frequency, respectively. By solving Equation 1, we obtain the driven mode amplitude 〈*Q*〉 = 〈*Q*〉_0_*e*^−*γt*^ sin(*ωt*) with


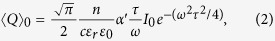


where *ω*^2^ = Ω^2^ − *γ*^2^, and in the small damping limit *ω* ~ Ω (337 cm^−1^ here). Although the phonon mode is not effectively driven by the laser pulse owing to the off-resonance condition, the mode amplitude has its maximum at the pulse duration time of 
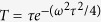
 in Equation 2, which has its maximum at 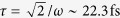. The number of optical cycles within this duration is assumed to be larger than 10, *i.e.*, a laser with frequency *f* > 448 THz should be used. Here we assume the working frequency is *f* = 600 THz. Using a value for *α*′ = ∂*ξ*/∂*Q* obtained from our DFT calculations and experimental values for the dielectric response *ε*_*r*_, we predict that the critical *Q*_3_ mode amplitude (*Q*_*c*_ ~ 0.5 Å AMU^1/2^) can be obtained with a pulse intensity of ~6 × 10^13^ W/cm^2^ for a 22.3 fs duration (Supplementary Fig. 2).

**Figure 6:**
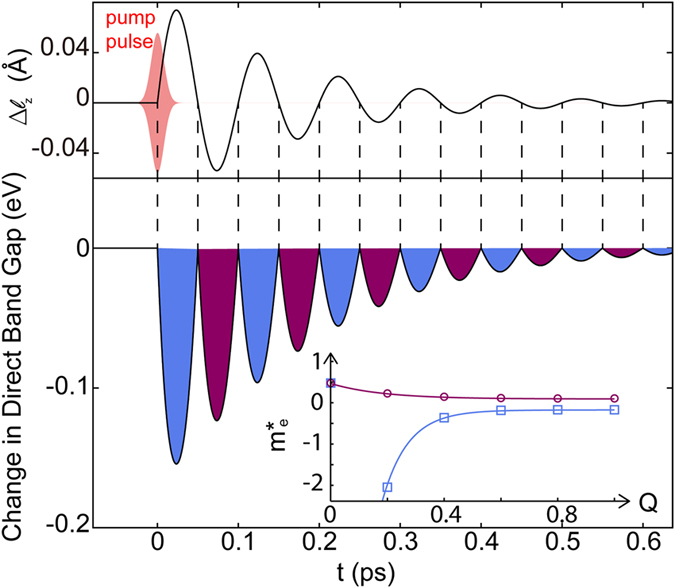
Dynamical structure-property relationships in LaTiO_3_. Dynamics of the phonon mode amplitude (upper panel) and the direct band gap change (lower panel) with time. For convenience, the phonon mode amplitude is converted to the difference in apical Ti-O bond length, which can be examined using synchrotron X-ray diffraction. The different colors of the direct band gap change denote the spin character of the valence band edge (spin up in blue, spin down in purple) in LaTiO_3_. Broken lines are guides-to-the-eyes to show that as the phonon modulation reverses its sign, the spin character of the valence band edge also flips, consistent with the time dependent oscillating spin-current described in Fig. 4. The inset shows the electron effective mass for the two spin channel as functions of the phonon mode amplitude.

Figure 6 shows the time-dependent mode amplitude, quantified in terms of changes in the apical Ti-O bond length, 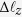 as defined in Fig. 2c, and the direct band gap following ISRS of LaTiO_3_. We find that the direct band gap is reduced by ~0.16 eV at the first maximum in the quasi-static *Q*_3_ phonon modulation, where the indirect-to-direct band gap transition occurs. During the second half of the oscillation period, the phonon modulation phase factor reverses its sign, which is shown as a negative value in 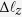. The minority (majority) spin Ti in the octahedra now undergo a 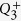 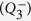 distortion. Therefore the composition of the valence band edge switches and is dominated by the minority spin states as indicated by the different shaded (colors) regions in Fig. 6. In the case of hole-doped LaTiO_3_ pumping the pure spin-current results in an alternating spin up and spin down carrier density at the picosecond timescale during coherent phonon excitation. Furthermore in LaTiO_3_, the effective masses for the two spin channels are the same at equilibrium, both positive (Fig. 6, inset); however, when the phonon mode is driven, the 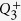 mode shifts the VBM energy at Γ. The electron effective masses 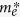 for the majority spin channels reverses its sign and then its magnitude gradually decreases, whereas the effective mass decreases with respect to the quasi-static amplitude of the normal mode in the minority spin channel.

#### Experimental Aspects of Band Engineering with ISRS

We now discuss the feasibility and technical issues for consideration to realize the proposed pump-probe ISRS experiment. First, photoelectrons and quasi-particle excitations are likely to occur in the ISRS process. To partially avoid some of these processes, the pump frequency should be selected to minimize absorption. Specifically for LaTiO_3_ we calculated the frequency-dependent optical absorption spectra (see Supplementary Fig. 3) and find that the proposed ISRS pump frequency (*f* = 600 THz = 2.5 eV) does not lead to severe electron excitation, which is consistent with the experimental optical conductivity spectra that shows no significant spectral weight for those energies^30^. When the experiment is carried out on the hole-doped case to explore the proposed ultrafast pure spin current, the optical absorption is slightly increased, however, still weak compared to the main absorption peak at *hv* = 6 eV. Also, quasi-particle effects may appear due to correlation as observed by the broad peak at ~2 eV in the DMFT spectral functions^31^. Owing to the far off-resonance condition in the ISRS process, the pumped dynamics is decoupled to the frequency of the pumping laser. One might also consider using a higher pump frequency around 4 eV, which would reduce both excitations.

Second, multi-photon processes might take place under the conditions of high laser intensity during ISRS. Although the centrosymmetric space group of LaTiO_3_ forbids the second order effect, third or high order processes are still possible but anticipated to be weak. Furthermore, since multi-photon absorption is a pure optoelectronic effect that often reduces frequency conversion efficiencies, it should therefore not qualitatively impact the phononic properties discussed here.

Third, the laser damage threshold (LDT) should be taken into account since the required pump laser intensity is high. Although the peak intensity is high, the pulse duration is short. A key parameter then to consider is the total fluence on the sample (here, 1.34 J cm^−2^). Since the LDT value for LaTiO_3_ is not well-documented, we use as an estimate that of LiNbO, which ranges from 10 to 22 J cm^−2^ and is one order of magnitude larger than the required laser fluence^32^. These material stability values and the fact that such laser fluencies have been achieved before support the experimental feasibility. Furthermore, a multi-pulse ISRS setup proposed in ref. 20 can be used to increase mode selectivity and thus the efficiency of the process, which is a possible way to reduce the laser intensity.

Finally, we note that the actual band gap of LaTiO_3_ is only 0.1~0.2 eV^33^. A band gap modulation may induce an insulator-to-metal transition owing to a collapse of the Mott gap, and an insulator-to-metal transition may occur before the indirect-to-direct band gap transition is observed. To explore this possibility, we examined the same dynamical band engineering processes with 1.4 < *U* < 4.4 eV. For *U* = 1.4 eV, the band gap is 0.25 eV which is close to the experimental value. The *Q*_3_ mode then induces an insulator-to-metal phase transition at an amplitude of 1 Å AMU^1/2^; at amplitudes below this value, the band structure is weakly modified. Upon a further increase in the amplitude of the *Q*_3_ mode from 1 to 3 Å AMU^1/2^, the induced metallic state transforms to a nearly semi-metallic state with a vanishing density of state at the Fermi level (Supplementary Fig. 4). After the mode amplitude exceeds 4 Å AMU^1/2^, the band structure is highly renormalized and screening effects quench the Coulomb potential induced by the FOJT distortion, leading to a spin degenerate AFM metal.

## Discussion

The proposed process here can be described as a light-induced Zeeman-like effect^34^ that may occur in any semiconducting AFM, making it applicable to materials beyond correlated oxides. The splitting due to the phonon deformation potential (*V*_def_) for the two spin channels^35^ can be written as a function of the nonequilibrium phonon amplitude. Keeping only the linear term, we have *V*_def_ = *μ*_*nk*_ × *Q*, where the coefficient *μ*_*nk*_ reflects the robustness of the transition and depends on the electron band *n* and wavevector *k*. For LaTiO_3_, *μ*_Γ_ ~ 0.5 eV Å^−1^ AMU^−1/2^ at the Γ point and similarly for CaMnO_3_, *μ*_*X*,*Y*,*Z*_ ~ 0.5 eV Å^−1^ AMU^−1/2^ at the **X**, **Y**, and **Z** points. In hole-doped LaTiO_3_, we predict a dynamically oscillating fully spin-polarized current, which is generated through the light-induced spin splitting at the VBM by coherent phonon excitations and it may be exploited for ultrafast spintronic devices. Our findings begin to uncover a key subtlety for the design of dynamic matter: The particular electronic configuration and the active frontier orbitals should be considered on equal footing with the symmetry of the targeted normal mode. By leveraging these criteria,we anticipate that control of the electronic band structures of solids at ultrafast timescales will usher in a new era of dynamical quantum materials.

## Methods

### Ab initio calculations and analysis

We perform first-principles density functional spin-polarized calculations within the revised generalized-gradient approximation exchange-correlation functional^36^ for solids by Perdew-Becke-Ernzerhof (PBEsol) as implemented in the Vienna *Ab Initio* Simulation Package (VASP)^37^,^38^ with the projector augmented wave (PAW) method^39^ to treat the core and valence electrons. The following electronic configurations are used in the calculation for LaTiO_3_: 6*s*^2^5*s*^2^5*p*^6^5*d*^1^ for La, 3*s*^2^3*p*^6^3*d*^2^ for Ti, and 2*s*^2^2*p*^4^ for O. For the calculation of CaMnO_3_ the following electronic configurations: 3*s*^2^3*p*^6^4*s*^2^ for Ca, 3*d*^6^4*s*^1^ for Mn, and 2*s*^2^2*p*^4^ for O. We sample the Brillouin zone using an 8 × 8 × 6 Γ-centered Monkhorst-Pack *k*-point mesh and integrations are performed using Gaussian smearing with the width of 0.01 eV. All structures are restricted to the observed orthorhombic *Pbnm* symmetry during structural optimization, whereby the lattice constants and atomic positions are relaxed until the stresses and forces on each atom are less than 3.5 kB and 0.1 meV/Å, respectively.

### Dynamical matrix calculations

The lattice dynamical properties are computed using the frozen phonon method, implemented in the PHONOPY software package^40^. After identifying the FOJT mode, the electronic band structure is calculated at different mode amplitudes under the quasi-static approximation. The displacement of atom *i* caused by the phonon mode is given by 
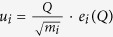
, where *u*_*i*_ is the displacement of the *i*th atom, *Q* is the normal mode amplitude, *m*_*i*_ is the atom mass of the *i*th atom, and *e*_*i*_(*Q*) is the atomic component of the eigenvector of the normal mode *Q*. The atomic displacements are scaled by a mass factor 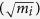. To facilitate straightforward experimental interpretation, a mode amplitude of 1 Å AMU^1/2^ changes the Ti-O bond lengths by 0.09 Å (apical) and 0.05 Å (equatorial), respectively.

### Wannierzation procedure

Since electron localization in a solid usually has an arbitrary orientation according to the local coordinate system, we use maximally localized Wannier functions (MLWFs)^41^,^42^ to analyze the orbital character of the localized electrons in the lower Hubbard band. Twelve *t*_2*g*_ bands are included in the projection, which span an energy window of −0.5–2.6 eV.

### Electron correlation analysis

Owing to electron-electron correlation effects among the 3*d* orbitals, we use the plus Hubbard *U* formalism following that of Dudarev *et al.*^43^, whereby a single *U*_eff_ parameter, hereafter *U* and discussed above, is used. For all calculations, we use a value of *U*(Ti) = 4.4 eV and *U*(Mn) = 5 eV based on previous constrained-LDA calculations^44^,^45^. First, this *U* value more closely reproduces the *a*/*b* axial ratio of LaTiO_3_, *i.e.*, 0.997 (Supplementary Table 1), which is in better agreement with the experimental value *a*/*b* ~ 1.008 for which DFT without a *U* correction predicts to be much smaller than unity. The octahedra rotation amplitudes show a different trend compared to the lattice constant; they are closer to the experimental value at low *U* values (Supplementary Table 1).

Second, owing to the local structural variations, we examined the dependence of the dynamical properties, specifically the phonon frequencies. Supplementary Fig. 5 shows the dependence of the phonon frequencies on the correlation strength *U*. We found that most mode frequencies are essentially non-dispersive with *U*, with the exception of the *Q*_3_ mode. It significantly softens with increased correlation as a consequence of electron localization onto a single orbital, which favors the nondegenerate state induced by the *Q*_3_ mode.

Last, as pointed out by others^31^, the magnitude of the band gap in the Mott state is something that should be considered alongside the structural parameters. Note that magnitude of the experiment LaTiO_3_ band gap is smaller than that obtained in our electronic structure calculations with the aforementioned *U* parameter; as Supplementary Fig. 6 shows, it can be tuned from approximately 0.25 eV to 2.0 eV upon increasing *U* from 1 eV to 4.4 eV, which also results in an orbitally polarized *t*_2*g*_ configuration. To examine the consequence of this dependence on the dynamic band engineering, we also evaluated the effect of *U* on the *Q*_3_ phonon modulated electron structure. Although the electron correlation changes the frequency of our selected phonon, we found no qualitative differences in the quasi-static structure–property interactions. The main effect is that the modulation in the direct band gap decreases with reduced correlation; for example, at *U* = 3.3 eV and a mode amplitude of 1 Å AMU^1/2^, the direct band gap is reduced to ~0.18 eV (Supplementary Fig. 6). The critical normal mode amplitude for the indirect-to-direct phase transition occurs at larger displacements.

Additional computational analyses of correlation effects and experimental discussions on the role of correlation and the FOJT phonon in LaTiO_3_ system can be found in refs.^31^, ^32^, ^46^ and 47.

### Impulsive Stimulated Raman Scattering (ISRS) analysis

Experimentally, one can excite the Jahn-Teller mode (*ω* = 337 cm^−1^) to exceed the critical amplitude (〈*Q*〉 ≈ 0.5 Å), and probe the band gap change with an ultrafast optical apparatus. Considering the symmetry of the Raman tensor for the *Pnma* space group, the polarization of the pump-laser should be parallel to the crystallographic direction [100] to drive the *B*_2*g*_ mode. The equation of motion of the phonon mode is given by Equation 1, where the impulsive driving force induced by the electric field of the laser can be written as 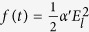 and *α*′ = ∂*α*/∂〈*Q*〉 is the derivative of the dielectric polarizability with respect to the amplitude of the specific Raman mode of interest. In practice, we compute this latter quantity at the DFT (PBEsol + *U*) level using a finite differences approaches. Note that the relaxation time appearing in Equation 1 depends on the damping strength *γ*, which can be measured in experiment; here, we use a typical value of 0.1Ω such that the damping constant is ~0.6 ps, which is long enough for detection by an ultrafast probe.

Now, suppose that the pulse has a Gaussian intensity distribution, such that


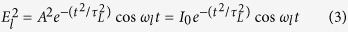


where *I*_0_ = *A*^2^ is the light intensity and *τ* is the pulse width. Since this process occurs at an ultrafast time scale, the frequency of the laser pulse is much higher than that of the phonon mode. In the following analysis, the number of cycles within one laser pulse width are assumed to be ~10 (see, Fig. 6a), giving 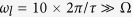. Thus, solution for the non-resonance limit is taken into account and the high frequency term *ω*_*l*_*t* does not drive the phonon mode effectively; therefore, it may be omitted.

The equation of motion is then solved as described in the main text to yield a driven mode amplitude defined in Equation 1. Since the phonon mode is not effectively driven by the laser frequency, mode selective excitation becomes a crucial aspect in design of a single pulse ISRS experiment. Nonetheless, it can be partially achieved by examining the expression for *Q*_0_. We find the duration time-dependence of *Q*_0_ from the term 
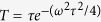
, which has its maximum at 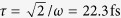. Therefore the excited mode can be maximized by controlling the pulse duration^18^. Such laser duration is now available with commercial Ti:sapphire lasers.

We use the following parameters for LaTiO_3_ to estimate the pump dynamics: The reflectivity for *ω* = 337 cm^−1^ is *n* = 0.5 (ref. 48), and the dielectric relative permittivity *ε*_*r*_ = 16.39 (ref. 49). This formalism with the approximated damping constant provides the mode amplitude as a function of time presented in Fig. 6b, and gives a required fluence of 1.34 J cm^−2^ which has been experimentally achieved before^50^,^51^.

## Additional Information

**How to cite this article**: Gu, M. and Rondinelli, J. M. Ultrafast Band Engineering and Transient Spin Currents in Antiferromagnetic Oxides. *Sci. Rep.* **6**, 25121; doi: 10.1038/srep25121 (2016).

## Supplementary Material

Supporting Information
