# Supplementary material for: Ultrafast Band Engineering and Transient Spin Currents in Antiferromagnetic Oxides
Source: Sci Rep. 2016 Apr 29;6:25121. doi: 10.1038/srep25121 (PMC4850389; doi:10.1038/srep25121)
Supplement: Supporting Information [file srep25121-s1.pdf]

SUPPLEMENTARY INFORMATION
FOR

Ultrafast Band Engineering and Transient Spin Currents in Antiferromagnetic Oxides

Mingqiang Gu and James M. Rondinelli*
Department of Materials Science and Engineering, Northwestern University, Evanston, IL 60208, USA

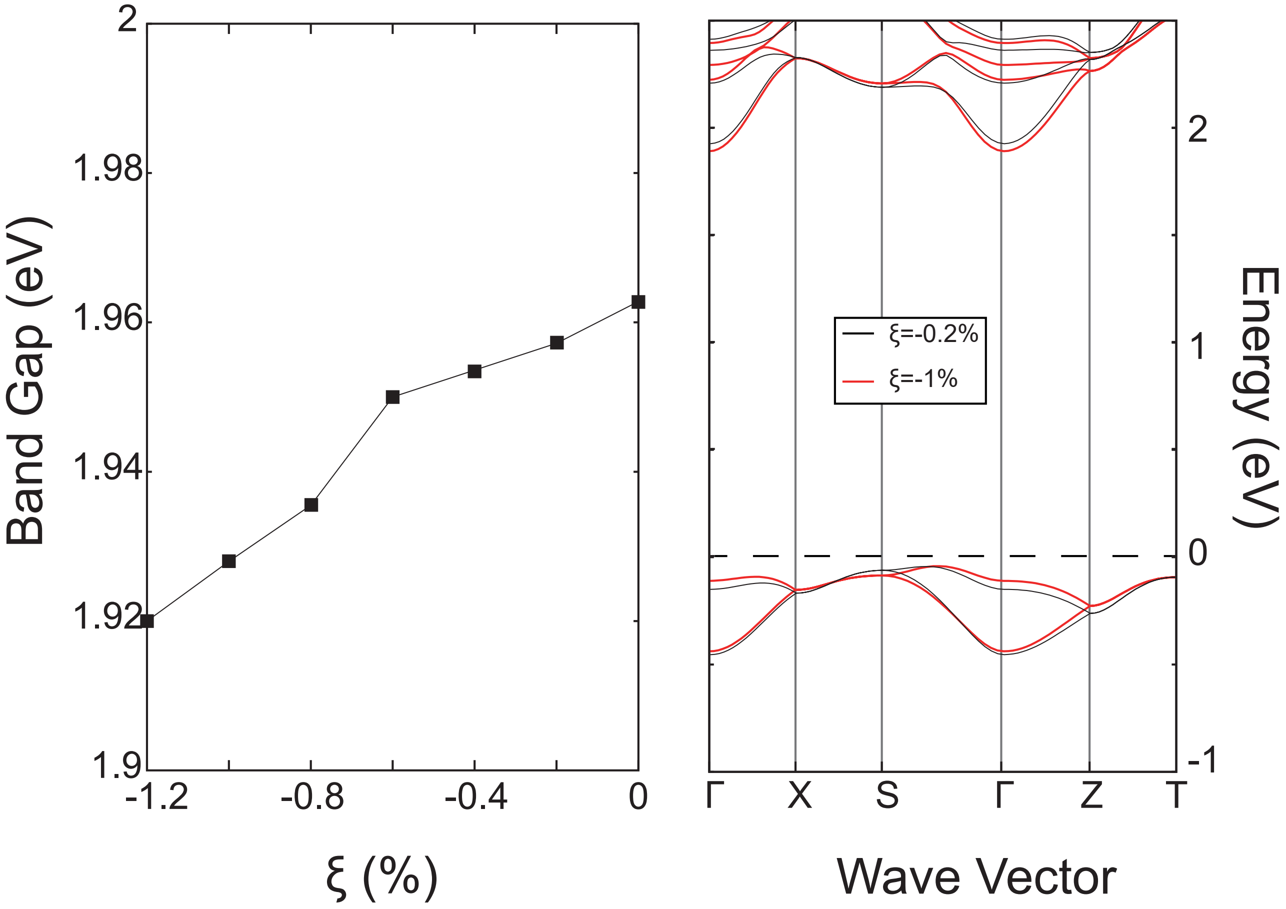

Supplementary Figure 1 | **Effect of static biaxial strain on the band structure of $LaTiO_3$.** (a) Band gap change as a function of the compressive strain ξ. Increasing compressive strain monotonically decreases the band gap linearly at a rate of 0.036 eV per percentage biaxial strain. (b) Band structure at two representative strain values. Compared to the Q_3 phonon modulation effects, two differences are noticed: (i) Strain does not lift the spin degeneracy, and (ii) Strain does not change the wave vector for the valence band maximum. Therefore, it is clear that the proposed phononic control of the electronic band structure is not accessible with biaxial (static) strain.

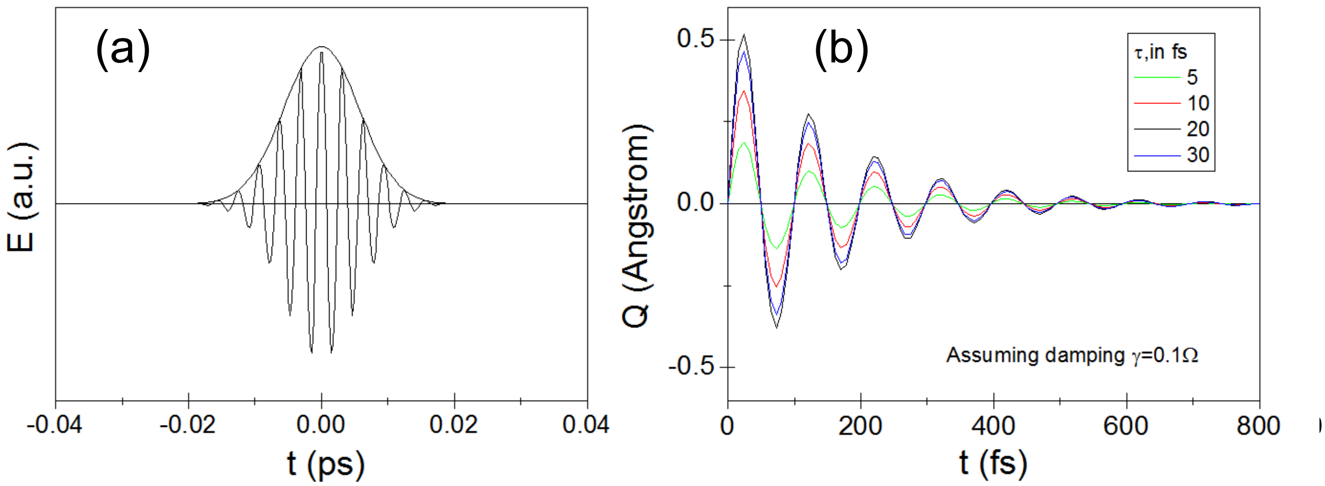

Supplementary Figure 2 | The dynamics of the pump pulse and the FOJT mode. (a) Schematic of the electric field as a function time for the ultrafast pump pulse. (b) Solution for the Q_3 mode amplitude, $\langle Q \rangle$, as a function of time for different pulse duration times with a pulse intensity $I_0 = 6 \times 10^{13}$ W/cm^2.

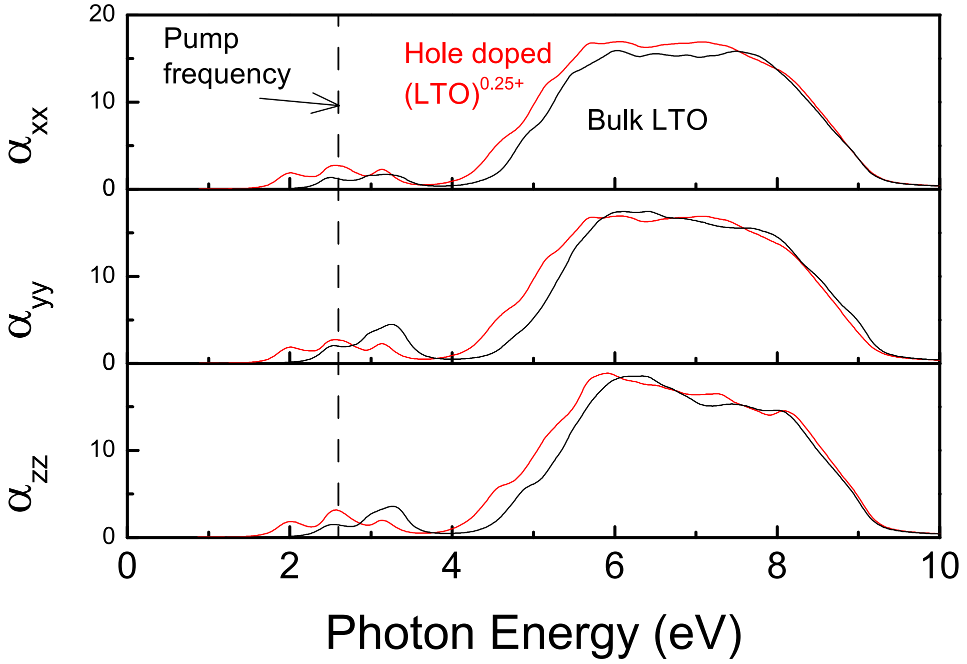

Supplementary Figure 3 | Optical absorption spectra for rare-earth titanates. The bulk $LaTiO_3$ (black) and hole-doped $(LaTiO_3)^{0.25+}$ (red) spectra calculated from DFT show that there is modest absorption at the pump frequency (denoted by the vertical line). The absorption could be further reduced by using a higher pumping frequency of $\sim$4 eV.

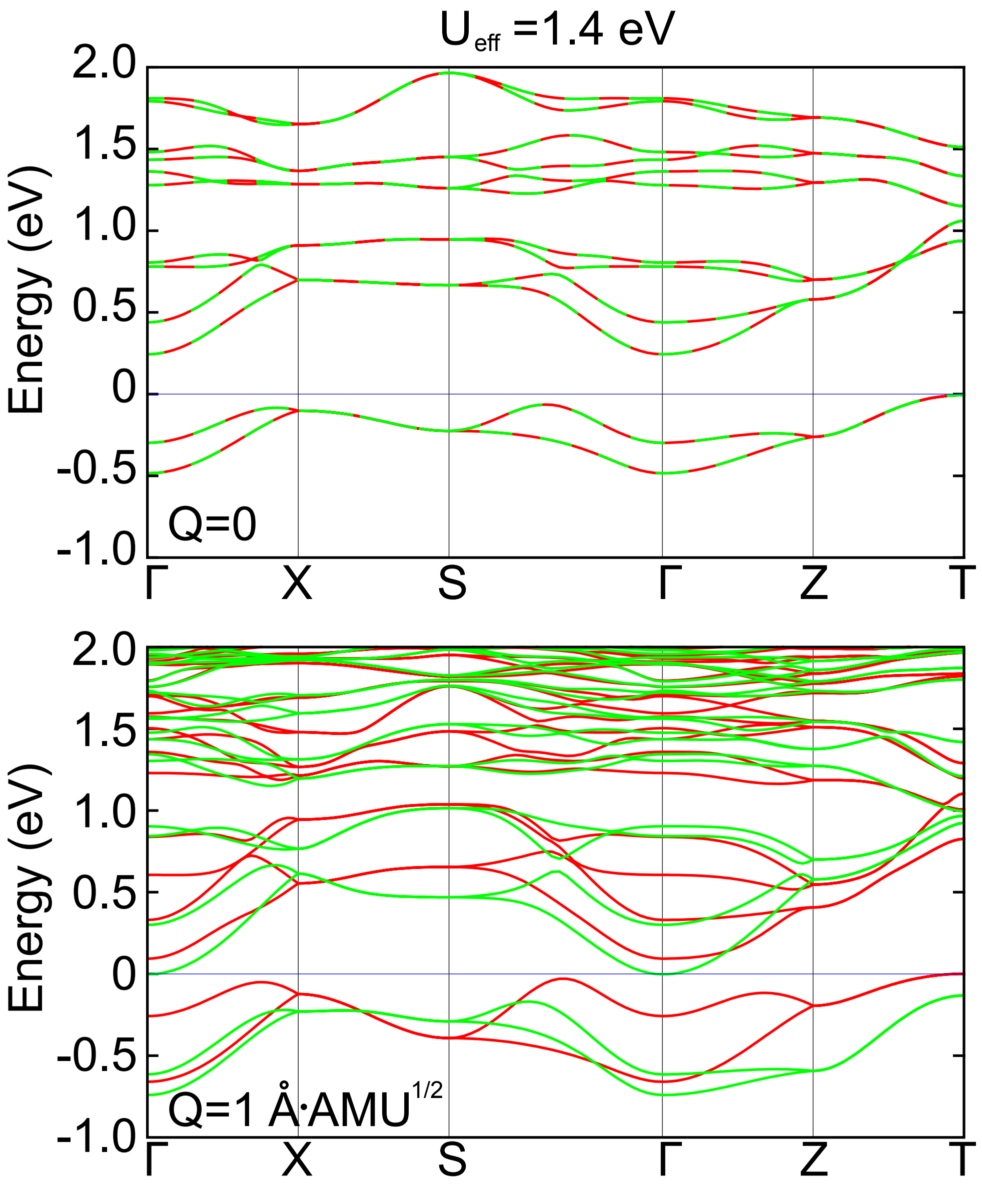

Supplementary Figure 4 | **Band gap collapse at a small value of U_{eff}.** The band structures for the equilibrium structure and for the phonon excited structure at $U_{\mathrm{eff}} = 1.4$ eV are plotted. Spin up and spin down channels are shown in red and green curves, respectively. We find that with a small value of U, the metal-insulator transition occurs before the indirect-to-direct band gap transition.

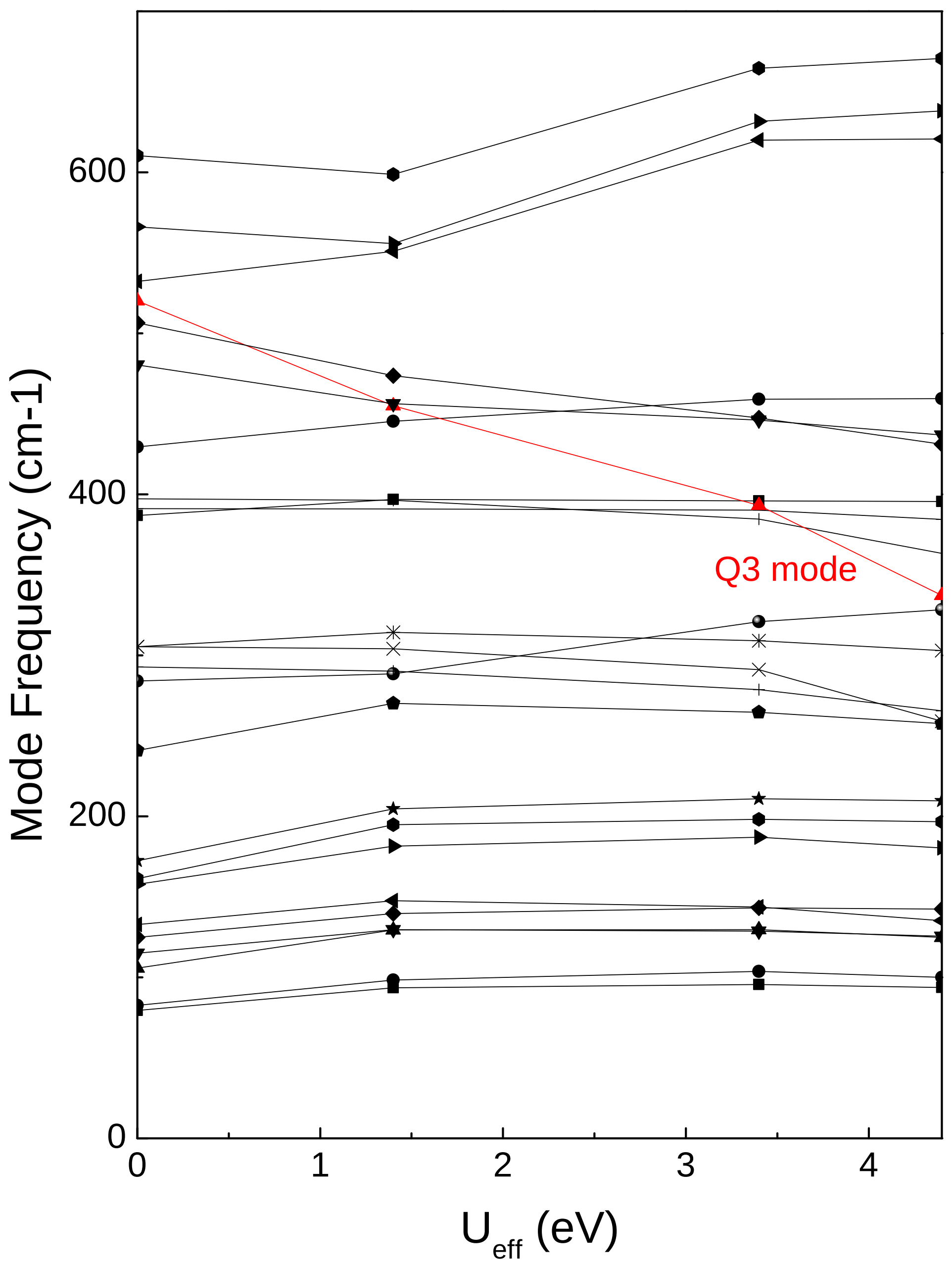

Supplementary Figure 5 | **Normal mode frequencies as functions of correlation.** The red curve corresponds to the Q_3 FOJT mode and strongly disperses with electron correlation (U_{eff}).

Supplementary Table 1 | Calculated crystallographic parameters for LaTiO$_3$. Data provided for orthorhombic LaTiO$_3$ ($Pbnm$, space group 62, Setting 3) using the PBEsol+$U_{\rm eff}$ functional compared to experiment. The Ti atoms occupy the $4b$ $(0,0.5,0)$ Wyckoff position without free parameters, while all other atoms have free paramaters as specified below: La and O(1) $4c$ $(x,y,1/4)$, and O(2) (x,y,z). The tilt $(180^\circ-\phi)/2$, rotation angles $(90^\circ-\theta)/2$ and the octahedra distortion index $\Delta=\frac{1}{6}\sum_i\frac{|l_i-l_{\rm av}|}{l_{\rm av}}$ are also listed. Here ϕ is the Ru-O$_{\rm apical}$-Ru bond angle, θ is the O-O-O angle between two neighboring basal TiO$_6$ octahedra, l_i is the ith Ti-O bond length within an octahedron, and $l_{\rm av}$ is the average length of the six Ti-O bonds.

	DFT-PBEsol+$U_{\rm eff}$				Experiment (10 K)
	$U_{\rm eff}$=1 eV	$U_{\rm eff}$=2 eV	$U_{\rm eff}$=3 eV	$U_{\rm eff}$=4.4 eV	Ref. 2
a (Å)	5.50	5.63	5.65	5.65	5.6301
b (Å)	5.59	5.60	5.63	5.67	5.5844
c (Å)	7.85	7.87	7.89	7.92	7.901
La	$x=0.99, y=0.06$	$x=0.99, y=0.06$	$x=0.99, y=0.06$	$x=0.99, y=0.06$	$x=0.99, y=0.05$
O(1)	$x=0.08, y=0.49$	$x=0.08, y=0.49$	$x=0.09, y=0.49$	$x=0.10, y=0.49$	$x=0.08, y=0.49$
O(2)	$x=0.71, y=0.30$	$x=0.71, y=0.30$	$x=0.71, y=0.30$	$x=0.70, y=0.30$	$x=0.71, y=0.29$
	$z=0.04$	$z=0.04$	$z=0.05$	$z=0.05$	$z=0.04$
Tilt Angle (°)	13.07	13.69	14.39	15.42	12.74
Rotation Angle (°)	9.53	10.01	10.23	10.56	9.17
Δ ($\times10^{-4}$)	0.00544	0.00673	0.00714	0.00844	0.00268

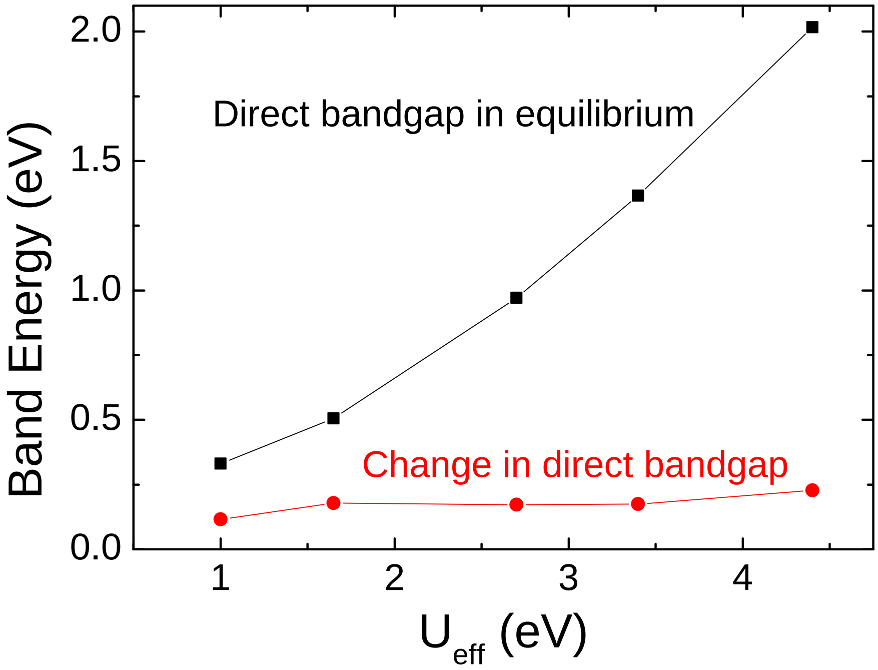

Supplementary Figure 6 | Correlation dependence of the band gap. The direct bandgap (black) in equilibrium and its change (red) when $Q = 1$ Å·AMU$^{1/2}$ as a function of U_{eff}. The change in the direct band gap is in the range from 0.17 to 0.28 eV.

References

---

* jrondinelli@northwestern.edu
[1] Betts, D. S., Maeno, Y., Awaji, S., Matsumoto, H. & Fujita, T. Lt-19transport and magnetic properties of laxsr1-xtio3. *Physica B: Condensed Matter* **165**, 1185–1186 (1990).
[2] Eitel, M. & Greedan, J. E. A high resolution neutron diffraction study of the perovskite $LaTiO_3$. *Journal of the Less Common Metals* **116**, 95–104 (1986).
